# Two new species of *Yunnanomonticola* Telnov (Coleoptera, Anthicidae) from China

**DOI:** 10.3897/zookeys.842.30741

**Published:** 2019-05-07

**Authors:** Yu-Chen Zhao, Zhang-Xun Wang, Xin-Pu Wang

**Affiliations:** 1 School of Agriculture, Ningxia University, Yinchuan, 750021, China; 2 Institute of Plant Protection, Shanghai Academy of Landscape Architecture Science and Planning, Shanghai, 200232, China

**Keywords:** Anthicidae, *
Yunnanomonticola
*, new species, China

## Abstract

Two new species of the genus *Yunnanomonticola* Telnov, 2002 are described based on the specimens collected in China. *Yunnanomonticolalatissima***sp. n.** is collected from Yunnan and *Y.tenuipenis***sp. n.** is from Guizhou. Photographes of the new species are provided, with a key to the three species of *Yunnanomonticola*.

## Introduction

**Figure 1.**
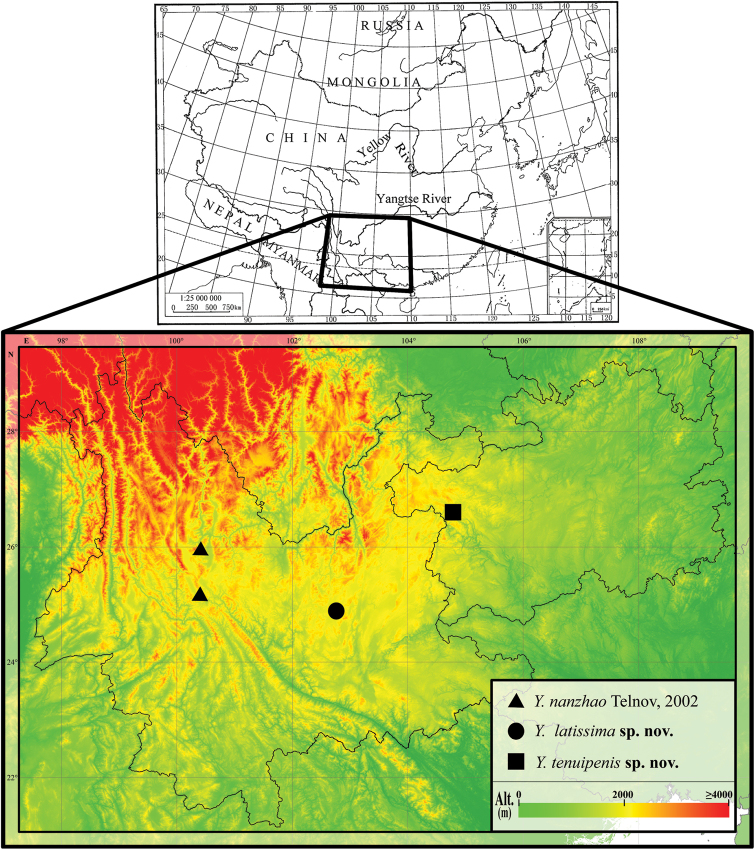
Geographic distribution of *Yunnanomonticola* species.

*Yunnanomonticola* was described as a monotypic genus for *Y.nanzhao* Telnov from Yunnan, China by Telnov (2002). Thereafter, no new species have been added to this genus. While examining Chinese specimens of anthicids collected from Yunnan and Guizhou provinces (Fig. 1), we identified two undescribed species of this genus. The purpose of this paper is to describe the two species as new in science.

## Material and methods

Specimens were collected by sweep nets or by hands and preserved in absolute ethanol. Male genitalia were examined after being cleared in hot 10% KOH solution. Specimens were examined with a Leica M205A stereomicroscope and measured with Leica Application Suite 4.12.0 software. Photographs of specimen were taken using a Leica DMC 4500 digital camera mounted on the stereomicroscope, photographs of anatomical structures were taken using Olympus DP27 digital camera mounted on Olympus BX53 biological microscope. Images of the same specimen at different focal planes were combined using Leica Application Suite 4.12.0 and edited with Adobe Photoshop CS6 and Macromedia Fireworks 8.0 software. Map based on SRTM 90 m Digital Elevation Data (http://srtm.csi.cgiar.org/), edited with ARCMap 10.0. The measurements and terminology follow Werner and Chandler (1995) and Chandler (2010).

Some anthicids can be attracted by cantharidin (Hemp and Dettner 2001). Cantharidin traps were tested in the field. To make the traps, specimens of *Mylabris* sp. (Meloidae) were dipped in pure ethyl acetate to extract cantharidin, the cantharidin was purified by absolute ethanol, then mixed with dried fine sand and preserved in Eppendorf tubules (5 mL).

All anatomical structures were stored in microvials with glycerol, which were pierced with the same pin as the body parts. Examined material, including the type specimens, were deposited in the Insect Collection, School of Agriculture, Ningxia University (SANXU), China.

## Taxonomy

### Yunnanomonticola latissima sp. n.

Taxon classificationAnimaliaColeopteraAnthicidae

http://zoobank.org/1E4BCA32-0FD0-4C07-9512-8127157CEFC0

Figs 2–7 , 14

#### Holotype.

♂, China, Mt. Xishan (24°56.26'N, 102°37.93'E), Kunming City, Yunnan, alt. 2,380 m, 11.VIII.2018, Yu-chen Zhao & Bing Yang. (Fig. 14)

#### Paratypes.

15♂♂, 13♀♀, same collection data as the holotype; 1♂, 2♀♀, Mt. Xishan (24°56.54'N, 102°37.90'E), Kunming City, Yunnan, alt. 2,390 m, 24.VII.2014, Zhang-xun Wang & Yong-qian Zhang.

#### Measurements, holotype.

Body length 2.30 mm. Head length 0.64 mm, maximum width 0.51 mm. Elytra length 1.10 mm, maximum width 0.67 mm. Eyes long axis 0.14 mm, short axis 0.11 mm. Pronotum length 0.65 mm, anterior lobe of pronotum maximum width 0.43 mm, minimum width 0.22 mm, posterior lobe of pronotum maximum width 0.30 mm.

#### Description.

***Color.*** Head, pronotum and elytra surface black. Pronotum with yellow margin basally. Femora and tibiae black except yellow at base of femora and at apex of tibiae; tarsi yellow, last segment darkened (dark form) or normal (pale form); claws slightly darkened. Antennae bicolor, antennomeres I–II yellow, III–V brown, rest antennomeres darkening to apex (dark form). In some specimens, antennae black, darkening to apex (pale form). Body black to blackish-brown in ventral view.

***Head.*** Oval, rounded basally, temporal angles absent, glossy. Eyes oval, small, convex. Frontoclypeal suture straight; labroclypeal membrane narrow, obscure. Clypeus with fine transverse wrinkles.

Vertex with large and shallow punctures, distance between adjacent punctures much smaller than their diameters, dense wrinkles present between antennae and eyes. Basal 2/5 of head almost smooth, with transverse punctures sparsely distributed, setation light colored, erect, longer than that on anterior head, pointing towards base of antennae. Head with setae of on anterior 3/5 shorter than setae on posterior head, suberect, pointing towards base of head. Setae of antennae bright, dense, fine and suberect; antennomere I equal to length of antennomeres II plus III, slightly wider than II and III; antennomeres II–IV equal in length, V–VII with same length, VIII–XI gradually increased in size; XI asymmetric, conical, 1.7 times as long as X. Gula smooth, less punctured than vertex of head. Terminal maxillary palpomere securiform, penultimate palpomere expanded inward. Neck ca. 1/4 time as width as head (include eyes), with shallow coarse punctures.

***Pronotum.*** Pronotum anteriorly with collar equally wide in dorsal and ventral views. Anterior lobe strongly convex in lateral view, glossy, with shallow median longitudinal groove, covered with short, light and suberect setae. Lateral margins of anterior lobe evenly rounded anteriorly, with small and sparse punctures. Pronotum strongly narrowed and constricted postero-laterally in dorsal view, with obvious longitudinal wrinkles at constricted area. Posterior lobe with two basal bumps, bearing small punctures unevenly spaced.

**Figures 2–7.**
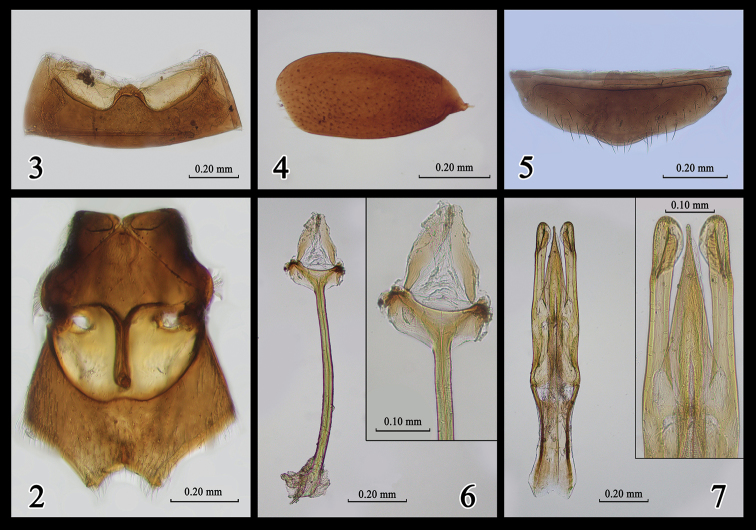
*Yunnanomonticolalatissima* sp. n., adult, male. **2** Meso- and metathorax, ventral view **3** sternum III, ventral view **4** left elytron, dorsal view **5** sternum VII, ventral view **6** spiculum gastrale **7** aedeagus, dorsal view.

***Thorax underside.*** Mesosternum with straight lateral margins, outer fringe of setae appressed to mesepisternum. Anterolateral margins of mesepisterna with fringe of long whitish setae. Lateral and distal parts of metasternum with long, dense, and subdecumbent pubescence (Fig. 2).

***Scutellar shield.*** Subtriangular, rounded apically, proscutellar with punctures, postscutellar smooth, nearly elongate ovate.

***Elytra.*** Glossy, lacking humeral angles, approx. half length of body, distal three abdominal terga usually not completely covered. Punctures fine and scattered, slightly denser at base and apex of elytra; distance between adjacent punctures ca. 2–8 times their diameters (Fig. 4); each puncture bearing one seta; pubescence dense at base (Fig. 14). Epipleura indistinct. Suture with distal 1/6 separated. Metathoracic wings reduced.

***Legs.*** Long and glossy. Setae on tibiae light colored, dense. Femora with distinct wrinkles. Metatibiae slightly bent inward. Tarsi with dense yellow setae, tarsomere I of posterior leg slightly curved, equal in length to sum of tarsomeres II–IV in length.

***Abdomen.*** Sternum III (first visible sternum) with dense punctures in middle (Fig. 3). Sternum VII simple in male (Fig. 5). Spiculum gastrale (sternum IX) thick, Y-shaped (Fig. 6).

***Aedeagus.*** Comparatively sclerotized, tegmen trilobed apically, median lobe tapering distally to pointed tip. Lateral lobes of tegmen symmetrical, thick and long, expanded apically (Fig. 7).

***Sexual dimorphism.*** Indistinct.

***Variation.*** Body length 2.04–2.71 mm (N = 29). Dark form: antennomeres I–II yellow, III–VI brown but lighter basally, rest segments gradually darkened to apex; apical of last tarsus black. Pale form: antennae black, gradually darkened from base to apex; last tarsus yellow.

#### Diagnosis.

*Yunnanomonticolalatissima* sp. n. differs from *Y.nanzhao*, as described by Telnov, by its mesosternum lacking obviously depressed pit (vs. mesosternum with six pit-like impressions medially), intercoxal projection of abdomen with lateral edges arched to apex (vs. straight lateral edges), median lobe of tegmen gradually tapering (vs. abruptly narrowed median lob), lateral lobes of tegmen thick (vs. thin).

#### Etymology.

The specific name comes from the Latin *latissimus* (wide), referring to the shape of the aedeagus.

#### Habitat and bionomics.

This species was found on the meadow near the top of a mountain at an altitude of ca. 2,380 m. Adults were clustered, active and crawling along the perennial gramineous plants and their tufted litters. Adults were not attracted to cantharidin traps.

#### Distribution.

China (Yunnan).

### Yunnanomonticola tenuipenis sp. n.

Taxon classificationAnimaliaColeopteraAnthicidae

http://zoobank.org/863D10B8-849D-49D0-BBDA-B01A4471FF5E

Figs 8–13 , 15

#### Holotype.

♂, China, Aogou Village (26°40.08'N, 104°48.44'E), Liupanshui, Guizhou, alt. 1,850 m, 14.VIII.2018, Yu-chen Zhao & Bing Yang. (Fig. 15)

#### Paratypes.

2♂♂, same data as holotype.

#### Measurements, holotype.

Body length 2.19 mm. Head length 0.55 mm, maximum width 0.46 mm. Elytra length 1.18 mm, maximum width 0.68 mm. Eyes long axis 0.12 mm, short axis 0.10 mm. Pronotum length 0.58 mm, maximum width 0.38 mm, minimum width 0.22 mm, posterior lobe of pronotum maximum width 0.28 mm.

#### Description.

***Color.*** Head and elytra surface black to blackish-brown. Pronotum blackish-brown, basal margin white, slightly yellowish. Femora and tibiae blackish-brown, lighter at apex of tibiae; tarsi yellow, apical segment darker at apex; claws yellow. Antennal color becoming darker from base to apex. Body blackish-brown on ventral side in ventral view.

***Head.*** Oval, rounded basally, temporal angles absent, glossy. Eyes oval, small sized, convex. Frontoclypeal suture straight. Labroclypeal membrane narrow, obscure. Clypeus with fine transverse wrinkles.

Vertex with irregular slightly shallow punctures, distance between adjacent punctures 0.3–1.0 times their diameters, with dense wrinkles between antennae and eyes. Basal 2/5 of head nearly smooth, with a few transverse punctures. Setation light colored, erect on basal 2/5 of head, pointing towards base of antennae. Setae on apical 3/5 of head suberect, shorter than setae on posterior head, pointing towards base of head. Antennomere III longer than preceding one, segments IV–VI being of equal length, VIII–X same in length; XI asymmetric, conical, 1.5 times as long as X; setae of antennae bright, dense, fine and suberect, VIII–XI with normal and very short setae. Gula smooth, less punctured than vertex of head. Terminal maxillary palpomere securiform, penultimate palpomere expanded inward. Neck ca. 1/4 time as width as head (including eyes), with coarse shallow punctures.

***Pronotum.*** Pronotum anteriorly with collar equally wide in dorsal and ventral views. Anterior lobe strongly convex in lateral view, glossy, median longitudinal groove shallow, covered with short, light and suberect setae; lateral margins of anterior lobe evenly rounded anteriorly, with small and sparse punctures, strongly narrowed and constricted postero-laterally in dorsal view, with distinct longitudinal wrinkles at contracted area. Posterior lobe with two small basal bumps, bearing small punctures unevenly spaced.

**Figures 8–13.**
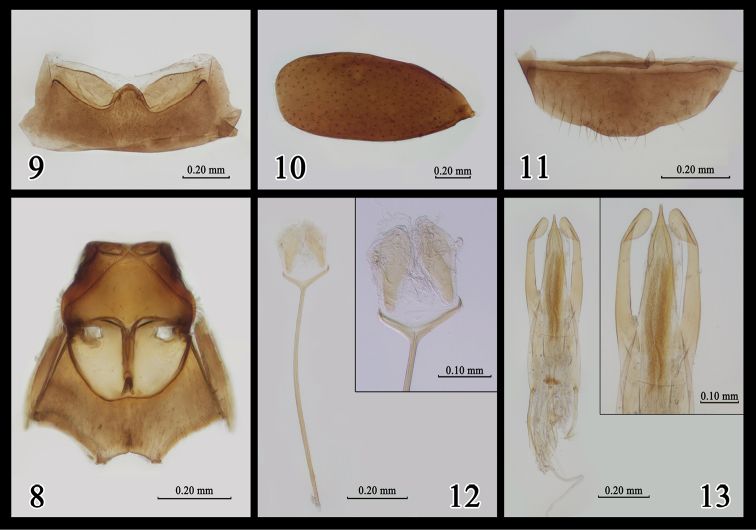
*Yunnanomonticolatenuipenis* sp. n. **8** Meso- and metathorax, ventral view **9** sternum III, ventral view **10** left elytron, dorsal view **11** sternum VII, ventral view **12** spiculum gastrale **13** aedeagus, dorsal view.

***Thorax underside.*** Mesosternum with lateral margins slightly bowed anteriorly, outer fringe of setae appressed to mesepisternum. Anterolateral margins of mesepisterna with fringe of long whitish setae. Lateral and distal parts of metasternum with long, separated, and subdecumbent pubenscence (Fig. 8).

***Scutellar shield.*** Subtriangular rounded apically, proscutellar with punctures, postscutellar smooth, elongate sub-ovate.

***Elytra.*** Glossy, lacking humeral angles, more than half of body length. Punctures scattered, evenly spaced, distance between adjacent punctures 4–6 times their diameter (Fig. 10). Pubescence dense in base and sparse in middle (Fig. 15). Epipleura indistinct. Metathoracic wings reduced.

***Legs.*** Long and glossy. Setae on femora and tibiae light colored. Femora with distinct wrinkles. Metatibiae slightly bent inward. Tarsomere I of hind leg with yellow sparse setae dorsally, slightly curved, equal in length to sum of tarsomeres II–IV.

***Abdomen.*** Sternum III (first visible sternum) with separated pubescence in middle (Fig. 9). Sternum VII simple in male (Fig. 12). Spiculum gastrale (sternum IX) thin, Y-shaped (Fig. 11).

***Aedeagus.*** Weakly sclerotized, median lobe of tegmen gradually narrowed towards apex, pointed apically; lateral lobes symmetrical, long, slightly swollen apically (Fig. 13).

***Variation.*** In dark form, femora and tibiae with uniform color. In some specimens, base of femora and apex of tibiae light yellowish-brown (at least in middle and posterior legs).

#### Diagnosis.

*Yunnanomonticolatenuipenis* sp. n. differs from *Y.nanzhao* by the scattered and evenly spaced punctures on the elytra (vs. punctures dense at base and apex), mesosternum with lateral margins slightly bowed anteriorly (vs. straight lateral margins), intercoxal projection of abdomen lateral edges arched to apex (vs. straight lateral edges), mesosternum lacking obvious pit-like depressions (vs. mesosternum with six pit-like impressions medially).

*Yunnanomonticolatenuipenis* sp. n. differs from *Y.latissima* sp. n. by its relatively longer elytra, relatively small bumps of pronotum, shorter and less dense pubescence of metasternum, shallower punctures of vertex, weakly sclerotized aedeagus, as well as elytra with fewer pubescence and distinct pattern of the punctures (vs. punctures dense basally and apically, sparse medially and laterally).

**Figures 14, 15.**
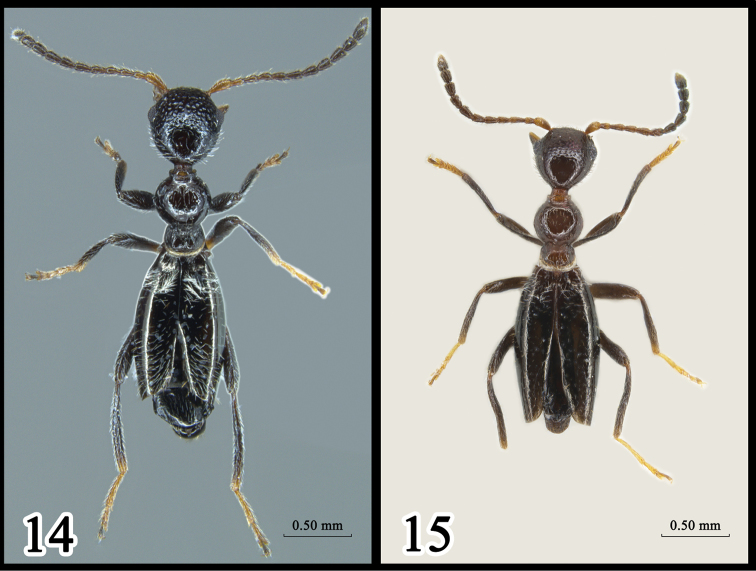
Adults of *Yunnanomonticola* spp. **14***Y.latissima* sp. n., holotype **15***Y.tenuipenis* sp. n., holotype.

#### Etymology.

The specific name is derived from the Latin prefix *tenui*- (thin) and “penis”, in reference to weakly sclerotized aedeagus.

#### Habitat and bionomics.

This species was found in grasses on the edge of woodlands at an altitude of ca. 1,850 m. Adults were clustered, active and crawling along the perennial gramineous plants and their tufted litters.

#### Distribution.

China (Guizhou).

## Discussion

*Yunnanomonticola* species are characterized by pronotum strongly narrowed and constricted postero-laterally in dorsal view, with two bumps at base; mesosternum with an outer fringe of setae appressed to mesepisternum; mesepisterna expanded laterally, with fringe of setae at the postero-lateral margin; mesepimera strongly reduced and indistinctive (Telnov, 2018); tegmen trilobed.

The genus *Yunnanomonticola* might be a montane species, as all three known species were collected in mountain areas above 1,800 m a.s.l. (Fig. 1) i.e., *Y.nanzhao* (2,000–2,800 m a.s.l.), *Y.latissima* sp. n. (2,380–2,390 m a.s.l.), and *Y.tenuipenis* sp. n. (1,850 m a.s.l.).

### Key to the species of the genus *Yunnanomonticola*

**Table:** 

1	Mesosternum with six pit-like impressions medially	***Y.nanzhao* Telnov**
–	Mesosternum lacking pit-like impressions medially	**2**
2	Elytra with dense punctures basally and apically, sparse punctures medially and laterally (Fig. 4); lateral lobes of tegmen and spiculum gastrale thick (Figs 6, 7)	***Y.latissima* sp. n.**
–	Elytra with evenly spaced punctures (Fig. 10); lateral lobes of tegmen and spiculum gastrale narrow (Figs 12, 13)	***Y.tenuipenis* sp. n.**

## Supplementary Material

XML Treatment for Yunnanomonticola latissima

XML Treatment for Yunnanomonticola tenuipenis
